# Factors that influence scope of practice of the five largest health care professions in Australia: a scoping review

**DOI:** 10.1186/s12960-022-00783-4

**Published:** 2022-12-23

**Authors:** Desmond Wiggins, Aron Downie, Roger M. Engel, Benjamin T. Brown

**Affiliations:** Department of Chiropractic, Macquarie University, Sydney, Australia

**Keywords:** Scope of practice, Scoping review, Australian health care, Health care professions

## Abstract

**Introduction:**

A well-functioning health system delivers quality services to all people when and where they need them. To help navigate the complex realm of patient care, it is essential that health care professions have a thorough understanding of their scope of practice. However, a lack of uniformity regarding scope of practice across the regulated health professions in Australia currently exists. This has led to ambiguity about what comprises scope of practice in some health care professions in the region.

**Objective:**

The objective of this review was to explore the literature on the factors that influence scope of practice of the five largest health care professions in Australia.

**Methods:**

This study employed scoping review methodology to document the current state of the literature on factors that influence scope of practice of the five largest health care professions in Australia. The search was conducted using the following databases: AMED (Allied and Complementary Medicine Database), CINAHL (Cumulative Index to Nursing and Allied Health Literature), Cochrane Library, EMBASE (*Excerpta Medica* Database), MANTIS (Manual, Alternative and Natural Therapy Index System), MEDLINE, PubMed, and SCOPUS. Additional data sources were searched from Google and ProQuest.

**Results:**

A total of 12 771 publications were identified from the literature search. Twenty-three documents fulfilled the inclusion criteria and were included in the final analysis. Eight factors were identified across three professions (nursing & midwifery, pharmacy and physiotherapy) that influenced scope of practice: education, competency, professional identity, role confusion, legislation and regulatory policies, organisational structures, financial factors, and professional and personal factors.

**Conclusion:**

The results of this study will inform a range of stakeholders including the private and public arms of the healthcare system, educators, employers, funding bodies, policymakers and practitioners about the factors that influence scope of practice of health professions in Australia.

**Supplementary Information:**

The online version contains supplementary material available at 10.1186/s12960-022-00783-4.

## Background

A well-functioning health system has been defined as one that ‘delivers quality services to all people when and where they need them’ [1]. In line with this description, the Australian healthcare system, which services approximately 26 million people, consists of a public and a private arm. The public arm operates via a federally funded ‘Beveridge’ model of universal health care insurance known as *Medicare*. The Beveridge model (established in 1948) is typically financed through a tax levy, which in Australia, is referred to as the ‘*Medicare* levy’ [2]. Currently the levy is set at 2% of an individual’s total taxable income [3]. *Medicare* provides all Australians with no or low-cost access to health and hospital services. The Beveridge model is currently utilised in several international jurisdictions including the UK, Spain, New Zealand, Scandinavia, and Iceland [4–6]. The private arm of the healthcare system is funded through premiums paid by members who take out private health insurance and covers some of the cost of treatment for private patients in private and public hospitals and can also cover some additional services not covered by the public arm.

The Australian health care workforce consists of regulated practitioners and unregulated health care workers (UHCWs), with the majority of care delivered by regulated practitioners within the public arm [7]. UHCWs in Australia are not registered under the National Scheme and have no mandated minimum education requirement, or defined scope of practice. Typically, they are employed in a variety of different primary care settings including aged and home care, general practice, community and correctional health care under the direction and supervision of registered health professionals (e.g. nurses) [8]. A similar scenario is reflected in Canada where older adults are assisted with personal support and the activities of daily living in a variety of care settings by UHCWs [9]. The absence of a defined scope of practice, combined with the lack of a mandated minimum education and baseline competency requirements in these jurisdictions, means that UHCWs may negatively impact quality of care and patient safety [9].

Prior to 2010, health care professions in Australia were regulated by profession-specific statutes that restricted practice and title [10]. More recently, the responsibility for providing title protection falls under the jurisdiction of the National Law [11]. However, there is currently a lack of agreement regarding what comprises scope of practice of Australian health care professions in the literature [12–15]. Furthermore, only one regulatory board has published a scope of practice guideline [16].

This approach has created a circumstance where individual practitioners are unsure about whether they are working within their profession’s scope of practice, who or what determines scope of practice, and if there are consequences (i.e. legal ramifications) associated with operating outside the profession’s scope of practice [17]. This uncertainty was seen in Australia during the COVID-19 pandemic with some states allowing health science students and certain health practitioners (e.g. physiotherapists) to operate outside their normal scope of practice by administering vaccines in a supervised setting after mandated training [18].

Given that 1 in 15 people employed in Australia is a registered health care practitioner [19], understanding scope of practice is critical [17, 20]. Uncertainty around the topic may negatively affect the ability of health care policymakers to formulate an appropriate skill mix as well as the distribution, recruitment, and retention efforts needed for care to be delivered when and where it is required. These issues are of particular importance in rural and remote areas that experience provider shortages. Furthermore, failing to define scope of practice means practitioners may contradict professional standards, risk patient safety, and violate national guidelines [21].

### Objective

The objective of this review was to identify factors that influence scope of practice of the five largest regulated health professions in Australia.

## Methods

Scoping review methodology was selected to collect and organise relevant information that addresses the study’s broad research question as well to provide an assessment of the current literature [22]. Unlike other reviews that typically focus on specific questions (e.g. systematic reviews), scoping reviews provide an overview of the developing evidence when it is uncertain what specific questions can be posed for evidence synthesis [22]. As the five largest regulated health care professions in Australia (nursing & midwifery, medical practice, physiotherapy, pharmacy, and psychology) comprise 86% of the combined regulated practitioners (*n* = 795 226 as of 2020) [19, 23], selection of literature around these professions was seen as being representative of all of the regulated professions (Table 1 lists all regulated professions).

**Table 1:** Professions regulated by AHPRA (2020)

Profession (as per AHPRA classification)	Number of registrants	Percentage
1. Nursing & midwifery	415 433^a^	56.0
2. Medical practice	125 641	16.0
3. Psychology	40 517	5.1
4. Physiotherapy	37 113	4.6
5. Pharmacy	34 512	4.3
6. Dental practice	24 406	3.0
7. Occupational therapy	23 997	3.0
8. Paramedicine	19 838	2.5
9. Medical radiation practice	18 243	2.3
10. Optometry	6 043	< 1
11. Chiropractic	5 777	< 1
12. Podiatry	5 608	< 1
13. Chinese medicine	4 921	< 1
14. Osteopathy	2 753	< 1
15. Aboriginal and Torres Strait Islander Health Practice	812	< 1
Total	801 659	100^b^

^a^This figure represents the total number of registrants in the NMBA, 409 000 Nursing, and 6 433 midwifery

^b^Due to rounding percentages may not total 100

Since the majority (98%) of registrants under the Nursing and Midwifery Board of Australia (NMBA) are nurses (*n* = 409 000/415 433 as of 2020) and the NMBA identifies nursing and midwifery as two separate professions [24], an argument could be made that nursing should be examined independent of midwifery. However, as the objective of the review was to examine the five largest health professions recorded under the jurisdiction of AHPRA (Table 1), we used the AHPRA classification (i.e. ‘nursing & midwifery’) and considered the two professions as one.

As AHPRA categorises the titles ‘enrolled nurse’, ‘registered nurse’, ‘midwife’, and ‘nurse practitioner’ under the term ‘nursing’ [25], papers referring to any of these professions were included. The Arksey and O’Malley’s five-step framework [26] and the Preferred Reporting Items for Systematic Reviews and Meta-Analyses Extension for Scoping Reviews (PRISMA-ScR) checklist were used to conduct this review [27–29].

### Five-step framework

#### Step 1: identifying the research question

A preliminary review of the literature revealed an absence of research on the scope of practice of the five largest health care professions in Australia. This led to the development of our broad research question: ‘What factors influence scope of practice of the five largest regulated health care professions in Australia?’ In this study, we defined ‘scope of practice’ as consisting of three distinct, dynamic, but interrelated components: ‘jurisdictional’ (legislative/regulatory), ‘professional’ (the profession); and ‘personal’ (individual practitioner). A description of the three interrelated components of scope of practice is presented in Table 2.

**Table 2:** Description of scope of practice components

Component	Description
1. Jurisdictional (legislative/regulatory)	Established on government practice acts that comprise regulations to safeguard patient safety [10, 30–32]
2. Professional (the profession)	Founded in a distinctive body of evidence, supported by educational preparation, and associated with a current or developing practice framework [30, 33, 34]
3. Personal (individual practitioner)	Focused on activities that an individual health care practitioner is educated and trained for, and that they can implement in a way that does not present any threat to the public or themselves [12, 17, 30, 33]

#### Step 2: identifying relevant papers

Peer-reviewed scientific literature and grey literature were identified through use of a search strategy developed in collaboration with a research librarian. The search included all papers published between January 2011 and May 2021. This date range was selected as the Council of Australian Governments (COAG) established the National Registration and Accreditation Scheme (NRAS) for health practitioners in late 2010 by introducing consistent legislation across all jurisdictions. Thus, 2011 was the first year dialogue around a national scope of practice could be undertaken [35]. The search strategy and indexing terms (MeSH and non-MeSH) relevant to the research topic used for each of the five professions can be found in Additional file 1. Google and ProQuest were used to retrieve grey literature relating to the research question.

#### Step 3: study selection

The review used the Joanna Briggs Institute (JBI) Population, Concept, Context (PCC) mnemonic. To be included in the study, an article had to relate to one of the five largest health care professions in Australia (Population), address a factor/s that influenced scope of practice for the salient health profession (Concept), relate to professional practice in Australia (Context), and be published in English between 2011 and 2021. Articles were excluded if they related to a country other than Australia, referred to another regulated (e.g. podiatry) or unregulated (e.g. Western Herbal Medicine) health profession, or were related to topics not pertinent to scope of practice. During the assessment process, if information relating to factors that could potentially influence scope of practice was not available in a study, it was deemed unsuitable for inclusion.

Initially, papers were screened by title and abstract to discover overtly relevant citations by two reviewers. The full-text version of each candidate citation was then retrieved and screened for eligibility by two reviewers. Any disputes were resolved by a third reviewer (BTB). All data (academic and grey literature) were managed using Clarivate Endnote^©^ and Microsoft Word^©^

#### Steps 4 and 5: charting, collating, summarising, and reporting study data

The data from eligible articles were charted: category, year of publication; author/s, country of origin, aim/purpose, method/study design, and influence/s (Additional file 2). An analysis was made of the research results including the number of papers included in the analysis, year of publication, and the study design. Each eligible paper was assessed for potential factors that influenced, defined, or formulated either scope of practice, advanced scope of practice, or expanded scope of practice, with each potential factor classified as a barrier or an enabler or both. Importantly, we did not determine a priori what the factors were that influenced scope of practice, rather we collected, then collated the factors that were reported in the literature.

## Results

The literature search yielded 12 771 publications. After removal of duplicates, 10 867 papers were screened; 10 836 articles were excluded leaving a total of 31 to be assessed for eligibility. Twenty-one articles met the eligibility criteria; two additional articles were discovered with forward/reverse citation tracking, making a total of 23 included articles: six literature reviews, five interviews, six surveys, one news item, two mixed method studies (consisting of a literature review and interviews), one editorial, and two government reports. No disputes arose during the assessment process (i.e. data extraction and screening). Twenty articles related to nursing, two to pharmacy, and one to physiotherapy. No articles relating to factors that influence scope of practice of medical practice or psychology were discovered. The number of papers included and excluded at each stage, along with the reasons for exclusion, are presented in a PRISMA flow diagram (Additional file 3).

### Reported factors that influenced scope of practice

Eight factors were reported in the included studies as influencing scope of practice. These were education [15, 36–49], competency [15, 36–38, 41–44, 46, 47, 50], professional identity [36, 51, 52], role confusion [15, 37, 39, 42, 44], legislation and regulatory policies [47, 50, 53, 54], organisational structure [36–38, 42, 44, 46, 55], financial factors [36, 37, 41, 42, 44–48, 52, 56, 57], and professional and personal factors [48, 52]. The factors identified in the literature have been reported verbatim, without interpretation or modification.

### Education

Several papers identified pre- and post-professional education as an enabler and/or barrier to scope of practice [15, 42, 43, 46, 47, 53, 58]. For example, Brown et al. [53] and Endacott et al. [39] found that when an enrolled nurse (EN) with a diploma-level qualification [59] completed post-professional education to transition to registered nurse (RN) (bachelor’s degree) [60], their scope of practice significantly increased. Their new responsibilities included undertaking wide-ranging patient assessment; developing a nursing care-plan in consultation with the multidisciplinary team and assessing the outcome; and administering medications and assessing the outcome. Additionally, Young et al. [46] discovered that post-professional, or ‘continuing’, education was an important influencing factor of nursing scope of practice, as it addressed skills that may not have been practised recently.

Furthermore, formal education, (‘organised and structured education with specific learning objectives’) and informal education (‘education with no set objective in terms of learning outcomes’) [61], were found to influence extended scope of practice, defined as the ‘discrete knowledge and skill base additional to the recognised scope of practice of a profession’ [36]. Similarly, Goodman et al. [40] revealed that post-professional education acted as an enabler to scope of practice of physiotherapy in regional emergency departments (EDs) in Australia. This led to the long-term sustainability of physiotherapy services in those settings.

### Competency

Eleven papers identified competency as a factor that influenced scope of practice of nursing in Australia [15, 36–38, 41–44, 46, 47, 50]. Two of these studies [42, 43] reported that competency influenced individual and/or personal scope of practice by acting as a quality assurance measure and reflecting the appropriate application of sound knowledge and skills within a particular vocational context.

### Professional identity

Rasmussen [51] reported confusion around professional identity had led to the absence of a clearly defined scope of practice for nursing in Australia, particularly in relation to child and adolescent mental health nursing. Hays et al. [52] reported that the need for services outside the usual medication management tasks had created a lack of clarity around professional identity of pharmacy within rural and remote Australian settings, and acted as both a barrier and an enabler to scope of practice. Other identified barriers were staff shortages, inadequate remuneration, and lack of further training, while the need for services, due to a lack of health providers in their area, and the capability of pharmacists to learn new skills and procedures were commonly identified as enablers to scope of practice [52].

### Role confusion

Five studies reported role confusion influenced scope of practice. For example, inconsistency in scope of practice language within professional codes and practice standards for nursing [15] combined with the development of new nursing roles [37, 44], created role confusion and hindered the formation of a well-defined scope of practice. Additionally, Endacott et al. [39] highlighted that the expansion of ENs’ scope of practice over the past decade had created an unclear function and competency differentiation between ENs and RNs which had led to role confusion and ongoing intra-professional debate. Furthermore, Jacob et al. [42] discovered that staff shortages and economic pressures created role confusion which influenced scope of practice.

### Legislation and regulatory policy

Several studies identified legislation and regulatory policy as factors that influenced the formation and application of scope of practice of nursing [38, 43, 46, 47, 62, 63]. For instance, Starr [62] noted that failing to act in accordance with government policy regarding scope of practice may lead to a disciplinary action, even if no adverse outcome occurred. Scanlon et al. [47] and Hains et al. [41] highlighted that scope of practice of nurse practitioners is influenced by Federal, State and Territory government legislative and regulatory requirements, i.e. post-endorsement authorisations. Moreover, it was found that because the changes to these requirements were not regularly circulated, scope of practice had been inadvertently limited [47]. Likewise, Puspitasari et al. [48] found that scope of practice of community pharmacists was influenced by government policies and regulations, particularly when pharmacists considered they were placed in situations where they had to ‘do a lot on the spot’. This development led to the perception that government policies portrayed pharmacists as dispensers of drugs, rather than health care professionals, a scenario that had the potential to jeopardise health care outcomes for patients [48].

### Organisational structure

Six studies and one government report identified organisational structures such as hospitals, as both a barrier and an enabler to scope of practice of nursing [36–38, 42, 44, 46, 55]. For example, Young et al. [46] and Queensland Health [36] found that the so-called internal ‘culture’ (e.g. a history of rigid and/or misconceived professional boundaries) within a structure was the most frequent reason for the lack of systemic implementation of scope of practice. Other factors were identified as a barrier to personal and legal scope of practice including: poor teamwork, high workload, lack of time to undertake assigned duties, high patient acuity, lack of supportive guidelines, fear of legal consequences for working outside of scope of practice, and workforce shortages [37, 38, 42, 44, 55]. Conversely, Birks et al. [38] found that organisational structures were an enabler to scope of practice as nurses often preferred to consult their peers and managers within the confines of the structure, rather than rely on professional and regulatory guidelines.

### Financial factors

A survey of nurses in Australia determined that the majority of participants (75%) believed insufficient financial reimbursement was a barrier to scope of practice [37]. Three studies found that insufficient remuneration, and/or economic factors associated with government schemes such as *Medicare*, particularly when combined with the absence of supportive guidelines on remuneration (i.e. reimbursable items) were barriers to scope of practice of nursing [37, 42, 56]. Similarly, Hays et al. [52] revealed that unsatisfactory remuneration for services was a common barrier to the scope of practice of pharmacists.

### Professional and personal factors

Two studies identified professional and personal factors as either a barrier or enabler to scope of practice of pharmacy, particularly in remote settings. Hays et al. [52] reported that the lack of other health services to refer to, limited number of clients, clients’ health beliefs and/or lack of motivation and expectations regarding the proposed treatment, lack of training for pharmacy staff, and time constraints were barriers to scope of practice.

Likewise Puspitasari et al. [48] identified that having to care for clients with specialised conditions could be a barrier to scope of practice of a community pharmacist due to insufficient pharmacist knowledge or a lack of patient motivation to engage with any additional support offered by the pharmacist. Conversely, having good patient rapport, job satisfaction and a positive attitude regarding their role as a health professional were identified as enablers to the formation of scope of practice of pharmacists [48].

## Discussion

The objective of this review was to identify factors (barriers and enablers) that influenced scope of practice of the five largest regulated health care professions in Australia. Eight factors were identified across three professions (nursing & midwifery, pharmacy, and physiotherapy): education, competency, professional identity, role confusion, legislation and regulatory policies, organisational structure, financial factors, and professional and personal factors. There is substantial crossover in the scope of practice of many of the AHPRA registered health care professions, e.g. occupational therapy and physiotherapy. While this review was restricted to the five largest professions in Australia, subsequent research could examine the scope of practice crossover amongst AHPRA regulated professions.

The lack of literature on the factors influencing scope of practice of medical practice in Australia is in contrast to international jurisdictions. For example, Russell et al. [64] discovered that four categories of influencers on scope of practice of medical practice exist within the US context: personal (e.g. training, work/life balance, mentoring); workplace (e.g. population, type of work, training); environment (e.g. proximity to hospital, isolation, health care regulations); and population (e.g. age demographics, bias toward speciality care, cultural norms regarding care). Additionally, Reitz [65] found that the broader health care landscape, local factors, and personal factors influenced scope of practice of medicine.

Within the Canadian context, Myhre et al. [66] identified that geographic factors (e.g. rural location, community size, distance to a large hospital), personal physician characteristics, professional education, and patient factors influenced scope of practice, while Kabir et al. [67] discovered that training, organisational structure, inadequate remuneration, workload, professional satisfaction, and the amount of patient care required per treatment influenced scope of practice. Additionally, Myles et al. [68] reported that geography, the practice environment, the needs of those within communities, and regional and jurisdictional variations in healthcare delivery were key elements in determining scope of practice of family physicians in Ontario, Canada. One influencing factor that was not discovered in the literature search that should be acknowledged is that of intra- and inter-professional issues.

### The importance of education in health care

Several Australian studies reported that pre- and post-professional education influenced scope of practice [15, 37, 42, 43, 46, 47, 53, 58] and facilitated the continuing development of the country’s healthcare system [40, 69, 70]. Studies from Canada offer further insight into the importance of education in health care, particularly ‘continued professional development’ (CPD). For example, Myles [71] and Horsley et al. [72] assert that scope of practice and CPD are inextricably linked, while Kam et al. [73] contend that education not only determines and maintains scope of practice over time, it is essentially the curriculum for CPD. In other words, education links scope of practice with CPD.

These findings suggest that health care professionals need to be well-educated and knowledgeable regarding advances in research and treatment modalities in order to practice in a competent manner [74]. This approach has been shown to assist in the discovery and application of health care approaches that help prevent disease and promote well-being [75, 76]. Importantly, the current pre- and post-professional education in Australia is reported as giving individual health care professionals the confidence needed to deliver quality health care within a full scope of practice [77, 78].

### The importance of competency

The core element of competency in Australian health care is the ability to practice in a manner that utilises critical thinking and accurate practice skills [79]. Any operational definition of competency must be straightforward and easy to understand as health professionals are required to adapt to changing clinical circumstances [80]. This approach is echoed in other jurisdictions [80]. Several papers identified competency as an influencing factor on scope of practice in Australian health care [15, 36–38, 41–44, 46, 47, 50], while international studies such as that from Kam et al. [73] highlighted that competency may be negatively impacted if a health care professional acts outside of their scope of practice. Several other influencing factors were reported including years of practice, age, certifications, and a workplace with a clear vision [81].

Other studies reported that competency acts as a quality assurance measure and reflects the appropriate application of sound knowledge and skills within a particular vocational context [42, 43]. These findings are congruent with the fundamental role of competency in health care, i.e. to execute care in a manner that generates a desirable outcome in the safest possible way [76]. This description suggests that competence is dynamic and changes over time [82]. In other words, a health care professional should be able to develop the ability to employ knowledge, skills, and abilities effectively to new settings, as well as to common tasks for which specified standards exist [83].

### The need for professional identity

The development of professional identity is a critical outcome of work-readiness programmes in health care (e.g. medical internships) [84]. It is a multifactorial phenomenon shaped by several factors including clinical and non-clinical experiences, motives, expectations, individual values, and beliefs and obligations [85]. Due to the influence of professional identity on scope of practice, interest in the topic has increased [86]. The term ‘professional identity’ appears regularly in the literature, but is typically ill-defined [85, 87]. To establish identity, health care professions often look for what is unique and different about their services in order to clarify identity and separate themselves from other professions [88].

Within the Australian health care setting, confusion around professional identity has led to the absence of a clearly defined scope of practice [51, 52, 89, 90]. Similarly, within the global context, scope and what constitutes professional identity appears to vary, despite extensive discussion on the subject [87]. For example, some researchers have defined scope of practice as a dynamic personal concept that develops from the commencement of pre-professional education, through to the health professional’s working life [91–95]. Others assert that professional identity comprises an integration of personal and professional values that must be internalised and committed to [96–98].

A clearly defined professional identity is important as it prevents scope of practice becoming more focused on roles that ‘fill gaps’, rather than retaining a paradigm-specific focus [99]. At the same time, it can reduce inconsistent and unsupported definitions of professional identity that often lead to misunderstandings and confusion amongst health care professionals [87], help prevent burnout, reduce loss of confidence in a profession, decrease role confusion [100–102], and more importantly develop a safe and effective scope of practice [15, 37, 51, 86, 103]. Therefore, it is imperative for a health care profession to have a strong professional identity otherwise the profession may have difficulty when considering its values and how they relate to the behaviours expected by the profession, colleagues, and the general public [101]. If the lack of clarity around professional identity is not ameliorated, patient safety may be jeopardised [86].

### Issues surrounding role confusion

Role clarity is crucial as poorly defined roles can become a source of conflict within clinical teams and reduce the effectiveness of care and services delivered to the population [104]. Several studies identified the existence of role confusion within Australian health care [15, 37, 39, 42, 44]. These studies suggested that a high level of standardisation of scope of practice [15, 37] combined with limiting role expansion can reduce role confusion [44]. The existence of role confusion causes concern to many health care professionals as it can potentially cause frustration, impede collaboration, create conflict, and constrain the improvement of knowledge and skills within a health care setting [105]. International studies assert that a well-defined professional identity, particularly within multidisciplinary settings [106], as well as a working knowledge of other health care professions’ roles, can help alleviate role confusion [39, 45, 107].

Contributing to these challenges are: legislative and regulatory frameworks that result in overlapping or encourage expanded scopes of practice; lack of clarity in workers' objectives; co-workers' expectations; the overall scope of responsibilities of their job; starting in a new organisation; a new supervisor or manager; a change in the structure of a work unit; and when a health care professional is required to perform a role that goes against their personal values [108]. A key challenge for all health care professions, not only in Australia but also globally, is to better define, differentiate, and demarcate the roles of each profession [42, 109–111].

### The influence of legislation on scope of practice

Specific principles outlined in legislation within the current Australian healthcare system can influence the scope of practice health care practitioners. Legislation aims to ensure that the highest quality of protection and care are afforded to the public [112], expedite access to services provided by health practitioners in harmony with the public interest and facilitate the continuous development of a flexible, receptive health care workforce [113]. These can be achieved, in part, by controlling what health care practitioners do through legislation [10].

Even though legislation is accepted as the “foundation of authority relevant to scope of practice” in Australia [50, 54], current legislation restricts scope of practice [47]. Moreover, jurisdictional, regulatory, and legislative changes that influence scope of practice often occur without broad consultation with the health care professionals delivering services. This scenario can lead to confusion around scope of practice [43, 47]. A recent US report highlights that the introduction of new regulations that seek to alter scope of practice are frequently costly, time-consuming and adversarial, due to an element of self-interest within the profession [114]. Conversely, legislation can be an enabler to scope of practice by providing role clarity for the profession [15].

### The influence of organisational structures on scope of practice

A healthcare structure is a place where patients needing a similar area of expertise are arranged into autonomously managed departments. Historically, the use of discrete healthcare structures was considered appropriate to support and foster the knowledge development necessary by medical science. More recently though, this framework has displayed considerable weaknesses including economic and organisational inefficiencies [115].

Australian studies shed light on this subject, highlighting that organisational structure can influence scope of practice [15, 42, 47, 116]. For example, the so-called ‘internal culture’ or ‘long-held traditions’ within an organisation can act as a barrier to scope of practice and limit improvement in health care access for the community [44, 46, 113, 117]. This attitude appears to disregard the fundamental purpose of a healthcare structure, to attain objectives that are outside the capacity of any single individual [118]. Similar results were reported in the US, where an entrenched ‘culture’ or ‘tradition’ within a structure may be a substantial barrier to scope of practice [119, 120].

### Financial factors

Several Australian articles indicate that scope of practice of health care professions is influenced by financial factors from two main areas: government funding [41, 48, 121, 122], and insufficient personal remuneration for services rendered [15, 36, 56]. The Medical Benefits Scheme (MBS), (a key component of *Medicare* Australia), can act as a barrier to scope of practice if MBS item numbers are limited for specific health services [41, 121, 122].

Halcomb et al. [57] found that as some government funding programmes operated on an ad hoc basis, subsequent opportunities for further development of scope of practice were limited. Moreover, insufficient personal remuneration (whether real or perceived) for services rendered can act as a barrier to scope of practice of health professionals in Australia [15, 36, 37, 56]. Two international reports support this view indicating that financial factors influence scope of practice of health care professionals [123, 124].

### The influence of professional and personal factors

Birks et al. [15] and Exercise & Sports Science Australia [125] highlight that scope of practice for health care professionals in Australia is influenced by professional and personal factors. Professional factors include practice environment, the need for a supportive working setting, personalised roles that are regularly revised and clarified, limited access to wider networks and geographic location [15, 56, 126]. Poor quality practice environments are a barrier to scope of practice as they typically engender unrealistic workloads, have poorly equipped facilities, and create unsafe working conditions. This makes it more difficult to entice, inspire and retain staff. Moreover, this setting reduces an organisation’s ability to meet performance targets [127].

Access to wider networks refers to the availability of other health professionals to refer to, while geographic location, in the context of these studies, refers to rural and remote settings. In the Australian context, legislative and regulatory provisions are in place across State, Territory and Federal governments to support the expanded scope of practice of novice and advanced health care professionals in rural and remote areas. For example, it is recognised that registered nurses need to be adequately prepared for the broader scope of practice necessary for rural and remote practice. Thus, educational programmes need to be flexible, accessible and affordable. Educational pathways should be structured to enable health care workers to expand their scope of practice according to the context in which they work and the needs of the community. A regular review of health legislation is needed to ensure there are no impediments to supporting advanced nursing practice within those settings [128, 129].

Rural and remote settings can be a barrier to scope of practice of some health care professionals because a lack of access to medical and/or specialised allied health staff can pressure some health professionals to work outside their normal scope of practice [15, 45, 49, 56, 126]. In other words, they perform tasks for which they have little or no training. This scenario is associated with higher levels of job dissatisfaction amongst Australian health care professionals [130] as individuals feel inadequately prepared for the extra responsibilities and experiential or technical challenges associated with an increased scope of practice [130]. In the global context, this is reflected in reduced role clarity which can jeopardise patient safety [131].

While professional and personal scope of practice are inextricably linked, it should be acknowledged that the scope of practice of an individual practitioner is distinct to the scope of practice of a profession. Personal factors that may influence scope of practice include being unable to undertake professional development, inexperience, stress, individual personality, motivation, and time constraints [56, 132]. Similarly, international studies demonstrate that personal circumstances such as time constraints, financial restrictions, and limited learning resources can influence scope of practice [133, 134]. Fundamentally, when workplace settings are optimised for a practitioner, health professionals tend to perform at peak scope of practice [135], leading to better health outcomes for patients.

### Strengths and limitations

A key strength of this review was the breadth of the literature search, which included multiple research databases and grey literature sources. Additionally, we compared scope of practice across multiple professions which, to our knowledge, has not been undertaken before in a single study. This is in contrast to other studies that typically focus on a single profession. A limitation of the review is that some articles may have been overlooked due to the constraints in our search strategy. For example, we used the terms ‘medical practice’ and ‘psychology’, but we did not look at the various sub-specialities within medical practice (e.g. plastic surgery and general surgery), or psychology (e.g. clinical psychology and forensic psychology). In addition, the paucity of Australian-based literature meant we were unable to systematically compare our findings across the five professions with other literature. Furthermore, the majority of studies included in this review related to nursing, which may limit the generalisability of our findings.

### Future research

The results from this review may serve to underpin future studies that investigate scope of practice for the remaining regulated health care professions in an attempt to identify if similar factors exist in those professions. Such studies may also help to determine if it is beneficial to have a common scope of practice across professions and whether this would assist in increasing competency and patient safety. Future research could also address several issues within the Australian context including: whether the regulated professions function equally well without a legislated scope of practice, or whether a formal, defined scope of practice is more acceptable for a profession and its patients. Furthermore, using the COVID-19 pandemic as a backdrop, future research could explore if the scope of practice of the largest health professions in Australia changed because of pandemic response measures and whether the pandemic impacted education, competence, and other factors identified in this study.

## Conclusion

Scoping review methodology was used to identify factors that influence scope of practice of the five largest regulated health care professions in Australia. Eight factors were identified across three professions (nursing, physiotherapy, and pharmacy): education; competency; professional identity; role confusion; legislation and regulatory policies; organisational structures; financial factors; and professional and personal factors. No studies were found for medical practice or psychology that met the inclusion criteria. While the role of each stakeholder group in health care is equally important, developing and implementing innovative approaches that provide appropriate context, structure, and definition of scope of practice of health care professions should be initiated by the respective education systems. The results of this study will inform a range of stakeholders including the public and private arms of the healthcare system, educators, employers, funding bodies, policymakers and practitioners regarding the factors that influence scope of practice of health professions in Australia.

## Supplementary Information

**Additional file 1.** The literature search strategy and indexing terms (MeSH and non-MeSH).

**Additional file 2: Figure S1.** PRISMA diagram.

**Additional file 3: Table S1.** Included papers.

## Data Availability

All data generated or analysed during this study are included in this published article and its Additional files.

## Abbreviations

AHPRA: Australian Health Practitioner Regulation Agency
COAG: Council of Australian Governments
ED: Emergency Department
EN: Enrolled nurse
EPS: Expanded pharmacy services
Govt.: Government
MBA: Medical Board of Australia
NP: Nurse Practitioner
NMBA: Nursing and Midwifery Board
NRAS: National Registration and Accreditation
Scheme OECD: Organisation for Economic Co-operation and Development
QH: Queensland Health
QLD: Queensland
QNU: Queensland Nurses Union
PHCO: Primary health care organisation
PHCOs: Primary health care organisations
RN: Registered nurse
SCOP: Scope of practice
UHCW: Unregulated health care workers
