# Supplementary material for: Factors that influence scope of practice of the five largest health care professions in Australia: a scoping review
Source: Hum Resour Health. 2022 Dec 23;20:87. doi: 10.1186/s12960-022-00783-4 (PMC9786531; doi:10.1186/s12960-022-00783-4)
Supplement: Supplementary file 1 — Additional file 1. The literature search strategy and indexing terms (MeSH and non-MeSH). [file 12960_2022_783_MOESM1_ESM.docx]

**Additional file 1: Literature search strategy**

**Databases**

AMED (Allied and Complementary Medicine Database), CINAHL (Cumulative Index to Nursing and Allied Health Literature), Cochrane Library, EMBASE (*Excerpta Medica* Database), MANTIS (Manual, Alternative and Natural Therapy Index System), MEDLINE, PubMed, and SCOPUS. Extra data sources were searched from Google and ProQuest.

**Indexing terms (MeSH and non-MeSH)**

A combination of terms relevant to the research were used with only the name of profession being changed. For example, scope AND practice AND nursing AND Australia OR barrier/s OR enabler/s OR influencer/s OR facilitator/s, scope AND practice AND physiotherapy AND Australia OR barrier/s OR enabler/s OR influencer/s OR facilitator/s and so on. Forward/reverse citation tracking was also undertaken on eligible papers.
