# Supplementary material for: Factors that influence scope of practice of the five largest health care professions in Australia: a scoping review
Source: Hum Resour Health. 2022 Dec 23;20:87. doi: 10.1186/s12960-022-00783-4 (PMC9786531; doi:10.1186/s12960-022-00783-4)
Supplement: Supplementary file 2 — Additional file 2: Figure S1. PRISMA diagram. [file 12960_2022_783_MOESM2_ESM.docx]

**Additional file 2: PRISMA diagram**

Records identified through database search
(n = 12,771)

## Identification

Records after duplicates removed

(n = 10,867)

## Screening

Records excluded

(n = 10,836)

- Did not match inclusion criteria

Records screened

(n = 10,867)

## Eligibility

Full-text articles assessed for eligibility

(n = 31)

Records excluded with reason:

(n = 10)

- Not related to factors that influence scope of practice of the 5 professions

Additional records identified with forward/reverse citation searching

(n = 2)

## Included

Records used in the study

(n=23)

**Figure S1**: PRISMA diagram
