# Supplementary material for: Factors that influence scope of practice of the five largest health care professions in Australia: a scoping review
Source: Hum Resour Health. 2022 Dec 23;20:87. doi: 10.1186/s12960-022-00783-4 (PMC9786531; doi:10.1186/s12960-022-00783-4)
Supplement: Supplementary file 3 — Additional file 3: Table S1. Included papers. [file 12960_2022_783_MOESM3_ESM.docx]

**Additional file 3: Table S1. Included papers**

**Table S1:** Included papers

| **Category** | **Year** | **Author/s** | **Country of Origin** | **Aim/Purpose** | **Method/Study Design** | **Type of Influence** |
| --- | --- | --- | --- | --- | --- | --- |
| **Nursing** | 2011 | Cant *et.al*. (45) | Australia | Explore rural RNs experiences while enrolled in post-professional education | Online survey | Education |
| **Nursing** | 2011 | QNU (50) | Australia | Define SCOP of nursing & nurses | Literature review | Legislation/regulatory policy  Education |
| **Nursing** | 2012 | Starr (64) | Australia | Understanding the importance of SCOP | Editorial | Legislation/regulatory policy  Education |
| **Nursing** | 2013 | Francis *et.al.* (57) | Australia | Identify barriers & enablers to advanced nursing in rural areas | Interviews | Personal factors  Organisational structure  Legislation/regulatory policy  Education |
| **Nursing** | 2013 | Jacob *et.al.* (42) | Australia | Present a history of ENs in Australia | Literature review | Competency standards |
| **Nursing** | 2014 | QNU (55) | Australia | Explain the relationship between clinical decisions and patient safety | News item | Legislation/regulatory policy |
| **Nursing** | 2014 | Qld. Health (36) | Australia | Identify how to increase allied health workforce SCOP in Queensland Health | Govt. report | Legislation/regulatory policy  Organisational structure  Financial factors  Education |
| **Nursing** | 2014 | Scanlon *et.al.* (47) | Australia | Explore legislative restrictions on NPs SCOP | Literature review | Legislation/regulatory policy  Education  Personal/professional factors |
| **Nursing** | 2015 | Rasmussen (51) | Australia | Identify framework of nursing practice with unclear SCOP | Interviews | Professional identity |
| **Nursing** | 2015 | Young *et.al.* (58) | Australia | Identify ways to expand allied health SCOP | Govt. report | Organisational structure  Legislation/regulatory policy  Financial factors |
| **Nursing** | 2015 | Brown *et.al.* (139) | Australia | Explore RN experience transitioning to RN | Interviews | Education |
| **Nursing** | 2016 | Birks *et.al.* (53) | Australia | Study SCOP of RNs in Australia | Literature review | Legislation/regulatory policy |
| **Nursing** | 2017 | King *et.al.* (43) | Australia | Determine role of PHCOs in supporting nursing profession | Mixed methods (literature review and interviews) | Legislation/regulatory policy |
| **Nursing** | 2017 | Halcomb *et.al.* (59) | Australia | Explore SCOP of Australian nursing | Online survey | Education |
| **Nursing** | 2017 | Murray-parahi *et.al* (49) | Australia | Compare RN & EN SCOP | Survey | Education |
| **Nursing** | 2018 | Birks *et.al.* (38) | Australia | Discover factors influencing SCOP of Australian RNs | Cross-sectional survey | Legislation/regulatory policy  Professional guidelines |
| **Nursing** | 2018 | Endacott *et.al.* (39) | Australia | Identify SCOP of ENs | Online survey | Education |
| **Nursing** | 2019 | Birks *et.al.* (37) | Australia | Report on barriers and enablers to expanding SCOP of RNs | Cross-sectional survey | Financial factors  Education |
| **Nursing** | 2019 | McKenna *et.al.* (44) | Australia | Report on a study of ENs SCOP development | Interviews | Organisational structure Education |
| **Nursing** | 2020 | Hains *et.al.* (41) | Australia | Compare international & Australian non-medical surgical assistant experience | Literature review | Legislation/regulatory policy  Personal/professional factors |
| **Pharmacy** | 2014 | Puspitasari *et.al.* (48) | Australia | Explore factors that influence SCOP for pharmacy practice | Semi-structured interviews | Personal/professional factors |
| **Pharmacy** | 2020 | Hays *et.al.* (52) | Australia | Identify barriers and enablers to SCOP for remote pharmacists | Cross-sectional survey | Professional identity |
| **Physiotherapy** | 2018 | Goodman e*t.al.* (40) | Australia | Determine how expanded SCOP can be enacted for physiotherapists in the ED | Mixed methods (literature review and interviews) | Organisational structure |

**Abbreviations:** RN: Registered nurse; SCOP: Scope of practice; EN: Enrolled nurse; PHCOs: Primary health care organisations; ED: Emergency Department
